# How Context Matters: A Dissemination and Implementation Primer for Global Oncologists

**DOI:** 10.1200/JGO.2015.001438

**Published:** 2016-01-20

**Authors:** Bogda Koczwara, Sarah A. Birken, Cynthia K. Perry, Deborah Cragun, Leah L. Zullig, Tamar Ginossar, Jesse Nodora, Neetu Chawla, Shoba Ramanadhan, Jon Kerner, Ross C. Brownson

**Affiliations:** **Bogda Koczwara**, Flinders University, Adelaide, South Australia, Australia; **Sarah A. Birken**, University of North Carolina at Chapel Hill, Chapel Hill; **Cynthia K. Perry**, Oregon Health & Science University, Portland, OR; **Deborah Cragun**, University of South Florida and Moffitt Cancer Center, Tampa, FL; **Leah L. Zullig**, Durham Veterans Affairs Medical Center and Duke University, Durham, NC; **Tamar Ginossar**, Department of Communication and Journalism and University of New Mexico Cancer Center, Albuquerque, NM; **Jesse Nodora**, University of California San Diego, San Diego; **Neetu Chawla**, Kaiser Permanente Northern California, Oakland, CA; **Shoba Ramanadhan**, Dana-Farber Cancer Institute and Harvard T.H. Chan School of Public Health, Boston, MA; **Jon Kerner**, Canadian Partnership Against Cancer, Toronto, Ontario, Canada; and **Ross C. Brownson**, Washington University in St Louis, St Louis, MO.

The need for a journal of global oncology reflects a growing realization by the oncology community that although evidence behind cancer control strategies may not differ around the globe, the context in which this evidence is applied varies according to available resources, societal values and priorities, policies, and health care systems.^1,2^ Context has profound influence on whether and how evidence is disseminated, adopted, tailored, and used in clinical practice. Context varies across countries, cultures, and geographic regions and reflects the great diversity of people affected by cancer. Recognition of and work within these diverse contexts may seem daunting, but the ability to identify and address the context within which we as clinicians and researchers practice is increasingly recognized as critical to making a lasting impact. The emerging field that explores how to apply evidence in different contexts often is called dissemination and implementation (D&I) science.^3^ Synonyms include knowledge translation, translational research, and implementation science.

This article, written by international cancer researchers with expertise in D&I science, explores the question of how D&I science can contribute to the advancement of global cancer control and the opportunities and challenges in this area. The concept for this article arose as a result of discussions during the 2nd Mentored Training for Dissemination and Implementation Research in Cancer convened at Washington University in St Louis in June 2015.^4^

## WHAT IS D&I SCIENCE?

D&I science addresses the sizeable gap between research and practice by purposefully and actively studying how to translate evidence into routine practice. Dissemination refers to the deliberate and active spreading of an evidence-based practice (EBP). Implementation refers to the process of translating EBPs into a specific setting.^5^ It takes an estimated 17 years for just 14% of original research to be disseminated and implemented in health care practice, even in countries with a relatively high resource base.^6^ This chasm between discovery and application of EBPs adversely affects outcomes across all fields of medical and public health practice.^7^ In cancer, the gap can have a profound impact. For example, the US Surgeon General issued a report that summarized the research on the impact of tobacco on health in 1964, yet decades passed before effective tobacco control programs were implemented.^8^ D&I science offers a potential to increase the likelihood and speed with which EBPs are successfully implemented by helping to understand and overcome many challenges posed by context variability.

## WHAT SHAPES THE CONTEXT OF CANCER CONTROL, AND HOW CAN PRACTITIONERS INFLUENCE IT THROUGH D&I SCIENCE?

Although sound evidence behind an EBP is fundamental to its dissemination and adoption, evidence alone is insufficient. Successful dissemination and implementation of evidence requires stakeholder engagement at multiple levels (eg, practitioners, consumers, payers), identifying and working with organizational champions, tailoring EBPs to meet organizational and patient characteristics and needs, and use of theoretical frameworks and models that clarify processes and factors that influence D&I.^9^ In contrast to clinical practice, D&I applies evidence at the system, organization, and individual levels rather than at the individual level alone. The sources of context variability described herein are common to the more than 60 frameworks and models that guide D&I research.^10^

### Economics, Resources, and Policy

Available resources, policies that drive resource allocation, and health system priorities all determine the magnitude of the push to adopt an EBP. Although availability of resources is a strong determinant of effective cancer control,^11^ many examples of innovative cancer control strategies in settings with modest resources exist (Table 1). A recent review conducted by *The Economist* of the quality of cancer control plans in Asia revealed that little correlation exists between quality of the national control plan and national resources, with one of the best plans reviewed coming from Thailand, a country of modest resources; countries of much more means like Japan and Australia, however, do not fare as well.^13^ One reason may be that policies can strategically distribute scarce resources due to stakeholder values and interests and promise of the EBP. This underscores the importance of engagement with key stakeholders, including policymakers, early in the course of planning for the implementation of EBPs.

**Table 1:**
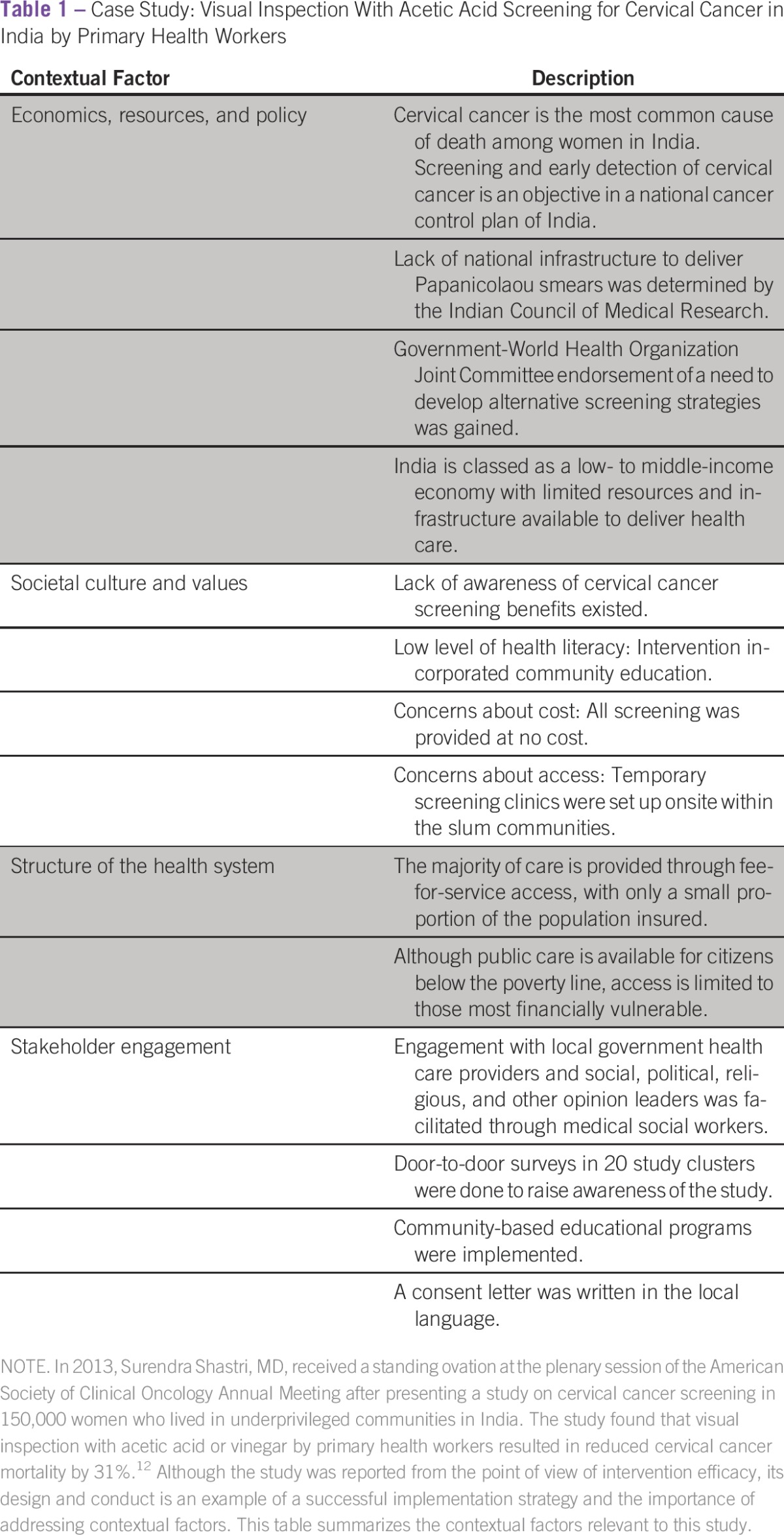
Case Study: Visual Inspection With Acetic Acid Screening for Cervical Cancer in India by Primary Health Workers

### Societal Culture and Values

Culture and values shape policy decisions, distribution of health care resources, and societal perception of need and desire to change. In this sense, societal culture and values may act as the pull of (or the push back against) EBP D&I. EBPs are more likely to be disseminated and implemented if they are perceived as consistent with user values and culture and have a relative advantage over current practices.^14^ Of note, in a complex setting, the relative impact of different factors varies. For example, although cultural values are often perceived as barriers to care (eg, cultural values of modesty among ultra-orthodox Jewish women are considered barriers to breast and gynecologic cancer screening), these are commonly associated with limited access to care and are successfully addressed when access is improved through system change, policy change, or resourcing.^15^ Successful implementation and meaningful stakeholder engagement require a recognition of the influence of culture and values in the context of the EBP in question.

### Structure of Health Care Systems

The health care systems within which care is delivered determine the fit influenced by the push and pull interaction among policies, resources, and societal culture and values. If the structure of the health care system aligns with the resources, policies, and culture and values (from which it is derived), the embedding of EBP into day-to-day operations is likely to be faster and more straightforward than when these factors present opposing forces. For example, survivorship care as a discipline is less prevalent in countries where the main priority is to provide adequate access to acute cancer treatment and the cancer advocacy organizations are in their infancy. In countries with well-developed and resourced health care systems with growing numbers of cancer survivors, the approach to delivering survivorship care varies according to how health care is delivered and funded. In nationalized health care systems (eg, United Kingdom, Canada), survivorship care continues to develop with an increased emphasis on delivery in primary care settings. In the United States, with a system largely comprising private payers and comprehensive cancer centers, most survivorship care remains in cancer care settings.^16^ Successful implementation of EBP requires consideration of how well the EBP can fit into the existing setting and whether its implementation requires manipulation of the push (policy), pull (stakeholder values), and/or change of fit through change of the health care system or setting.

### Stakeholders

Although the D&I process is undertaken mostly at the system rather than at the individual level, one cannot successfully engage, communicate, and negotiate with the system without engaging the people who represent various aspects of the system (eg, clinicians, patients, policymakers). In the ideal setting where the pull, push, and fit are just right, the engagement would consist of building common understanding, agreeing on expectations, and planning how to work together. In real life, these processes are achieved through negotiation. Stakeholder support is essential for successful implementation of EBPs and continues to be a key factor in the acceptability and sustainability of a program. Stakeholders’ differing goals, priorities, and engagement in change and differing levels of health literacy potentially affect their ability to make informed decisions. Stakeholder perspectives may vary dramatically, even in areas of geographic proximity and similar resources. For example, the Choosing Wisely campaign to deimplement ineffective practices in cancer care led to different priority lists in Canada and the United States and different approaches to how these lists were generated. In the United States, the Cost of Care Task Force listed five practices that should be avoided and presented it to the ASCO Clinical Practice Committee, advocacy groups, and the ASCO Board of Directors for endorsement. The list comprised specific practices, three of which were related to cancer tests for staging and follow-up.^17^ In contrast, the Canadian Partnership Against Cancer adopted a four-stage approach that refined 66 potential practices into 10, with only one overlapping with the ASCO list.^18^

## A CALL TO ACTION FOR GLOBAL ONCOLOGISTS

As oncologists become global practitioners, D&I science offers tools to broaden their clinical and research endeavors (Table 2). D&I science clarifies the challenges that stem from diverse contexts as well as the diverse factors that may aid practitioners’ efforts to adopt EBP to fit these contexts. Dedicated funding opportunities for D&I research through the National Cancer Institute, Patient-Centered Outcomes Research Institute, and other international grant funding schemes attest to the increasing recognition of the value of D&I by funders and policymakers, but its value must be equally embraced by the oncology community through integration of D&I methodology into clinical practice, research design, and evaluation of interventions and programs.

The oncology community must acquire new skills and recognize not only the content but also the context of their practices and design and report research with various contexts in mind. Likewise, the field of D&I must broaden its reach beyond its current geographic focus, which remains mostly within the United States, United Kingdom, Canada, and Australia. The ideal time is now to open the discussion about how to integrate the concepts of D&I into day-to-day practice of global cancer prevention and control. May that be the lasting legacy of the *Journal of Global Oncology*.

**Table 2:**
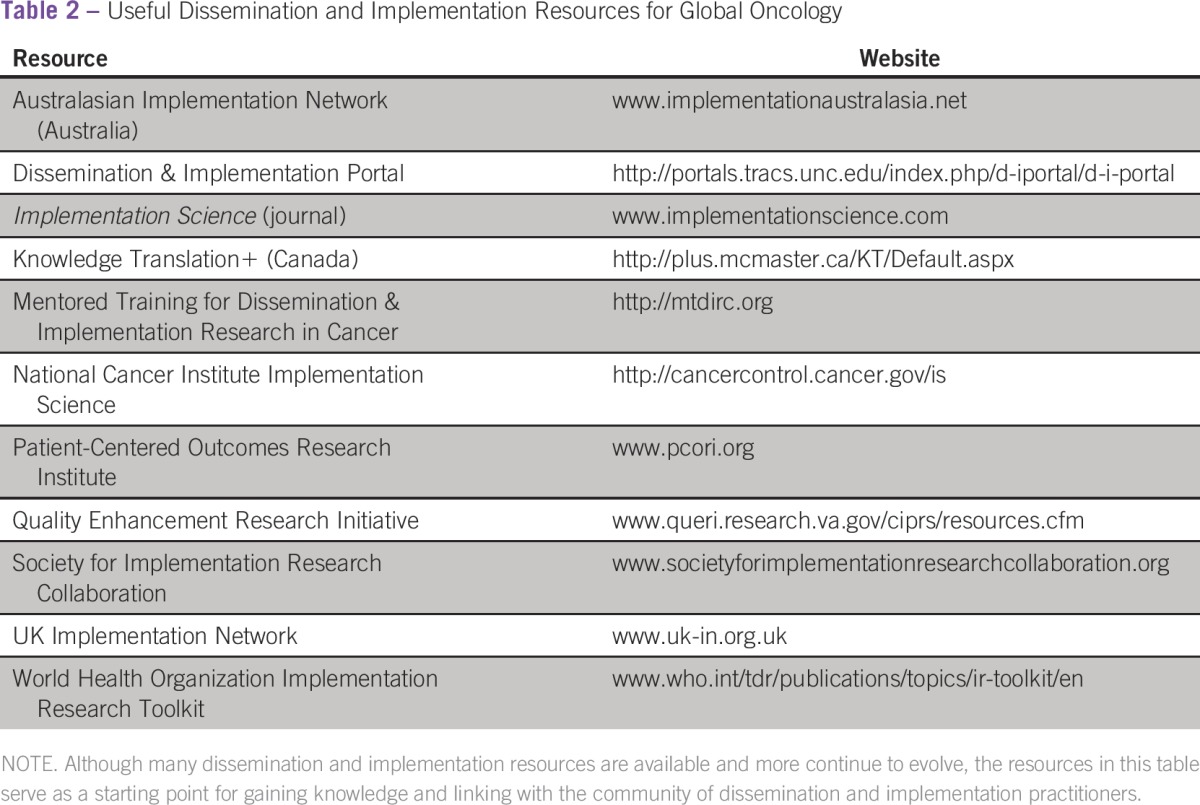
Useful Dissemination and Implementation Resources for Global Oncology
